# Mature NK-cell lymphoma presenting with chylothorax and delirium: a rare emergency case

**DOI:** 10.1186/s12245-025-01076-y

**Published:** 2025-12-29

**Authors:** Wen Shang, Zhiguo Guo, Qingbian Ma, Jijun Wang, Yuchang Xu

**Affiliations:** 1 Department of Emergency, Peking University Third Hospital, 49 North Garden Rd.,Haidian District, Beijing, 100191 P. R. China; 2 Key Laboratory of Molecular Cardiovascular Sciences, Ministry of Education, 49 North Garden Rd.,Haidian District, Beijing, 100191 P. R. China; 3 Department of Hematology, Peking University Third Hospital, 49 North Garden Rd.,Haidian District, Beijing, 100191 P. R. China

**Keywords:** Chylothorax, Delirium, Mature NK-cell tumor

## Abstract

**Background:**

Extranodal natural killer (NK)/T-cell lymphomas constitute less than 1% of non-Hodgkin lymphomas, with central nervous system involvement and chylothorax representing exceptionally rare presentations. This unique combination has not been previously reported in emergency medicine literature.

**Case presentation:**

A previously healthy 51-year-old Chinese male presented with progressive dyspnea, high fever, and bilateral pleural effusion over eight days. Physical examination revealed extensive ecchymoses, bilateral lung consolidation, and splenomegaly. Laboratory studies demonstrated pancytopenia (WBC 3.26 × 10⁹/L, platelets 58 × 10⁹/L), severe coagulopathy, and elevated lactate dehydrogenase (773 U/L). Thoracentesis confirmed chylothorax with triglyceride levels of 1.46 mmol/L and positive Sudan III staining. On hospital day 10, the patient developed acute hyperactive delirium with visual hallucinations and paranoid behavior. Flow cytometry revealed abnormal NK-cell expansion across peripheral blood (68.09%), bone marrow (13.75%), and pleural fluid (87.34%), establishing the diagnosis of mature NK-cell lymphoma with multi-site involvement. PET-CT imaging demonstrated splenic involvement (SUVmax 9.0) and a hypodense lesion in the left occipital lobe. Despite supportive care including oxygen therapy, empirical antibiotics, and sedation, the patient rapidly progressed to hemophagocytic lymphohistiocytosis and multi-organ failure, resulting in death within four weeks of presentation.

**Conclusions:**

This case demonstrates that mature NK-cell lymphoma may present with the previously unreported combination of chylothorax and acute delirium in emergency settings. Emergency physicians should maintain heightened awareness of hematologic malignancies when encountering patients with unexplained pleural effusion, neuropsychiatric symptoms, and cytopenias, as early recognition and prompt hematology consultation may influence clinical outcomes despite the typically aggressive disease course.

## Background

Extranodal NK/T-cell lymphoma represents a rare and aggressive subset of non-Hodgkin lymphomas with marked geographic variation, being significantly more common in Asian and Latin American populations [1, 2]. According to the 2022 WHO classification, these tumors are characterized by an activated NK-cell or cytotoxic T-cell phenotype with strong association to Epstein-Barr virus infection [1, 3]. While nasal presentations predominate, extranasal manifestations can involve diverse anatomic locations and often present diagnostic challenges in emergency settings [4, 5].

Chylothorax remains exceptionally rare in NK/T-cell lymphoma with only sporadic case reports in the literature [6, 7]. Similarly, central nervous system involvement is uncommon and typically manifests as focal lesions rather than diffuse neuropsychiatric symptoms [8]. The combination of chylothorax and acute delirium as presenting features has not been previously reported. NK/T-cell lymphoma may initially be misdiagnosed due to overlapping clinical presentations with infectious conditions, and such diagnostic delays can prove fatal given the tumor’s aggressive nature and propensity for rapid progression to hemophagocytic lymphohistiocytosis [9, 10].We present the first documented case of mature NK-cell lymphoma presenting simultaneously with chylothorax and delirium in an emergency department setting.

## Case presentation

**Fig. 1:**
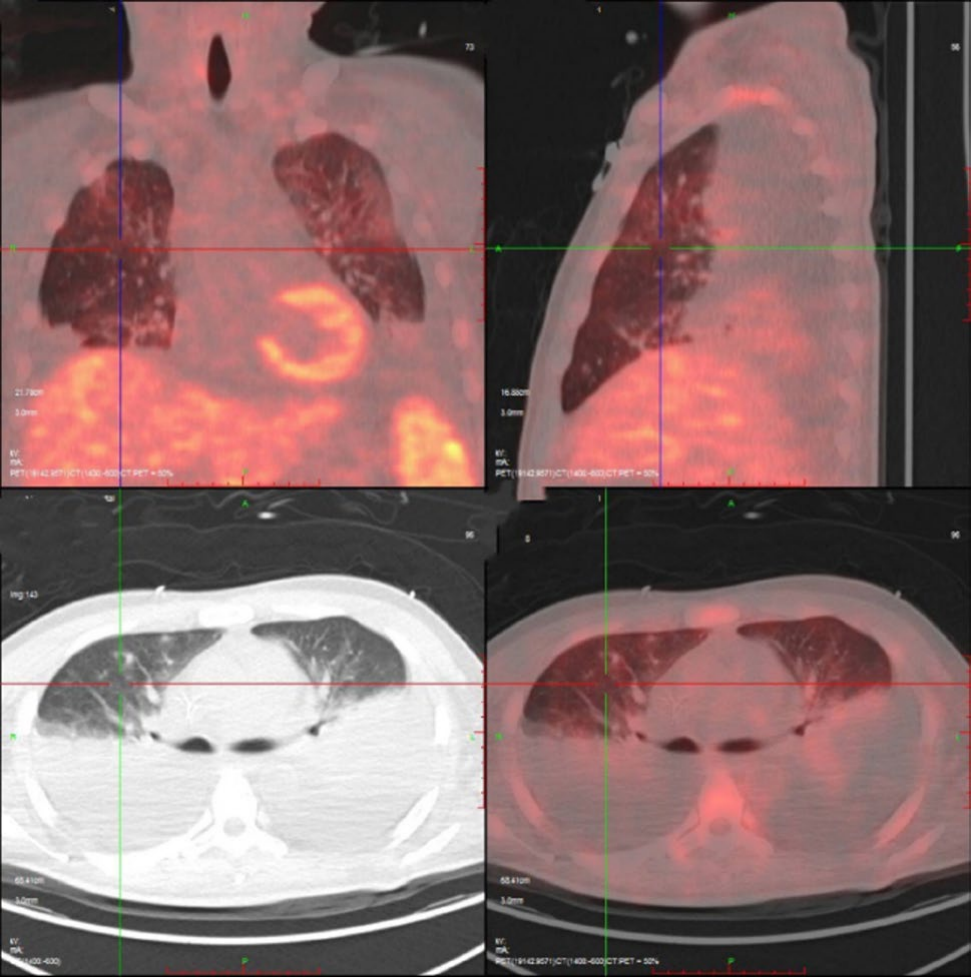
PET-CT imaging reveals bilateral pleural effusions with no abnormal FDG uptake within the pleural fluid. The absence of metabolic activity suggests a reactive effusion secondary to lymphatic obstruction rather than malignant pleural involvement, supporting the diagnosis of chylothorax

**Fig. 2:**
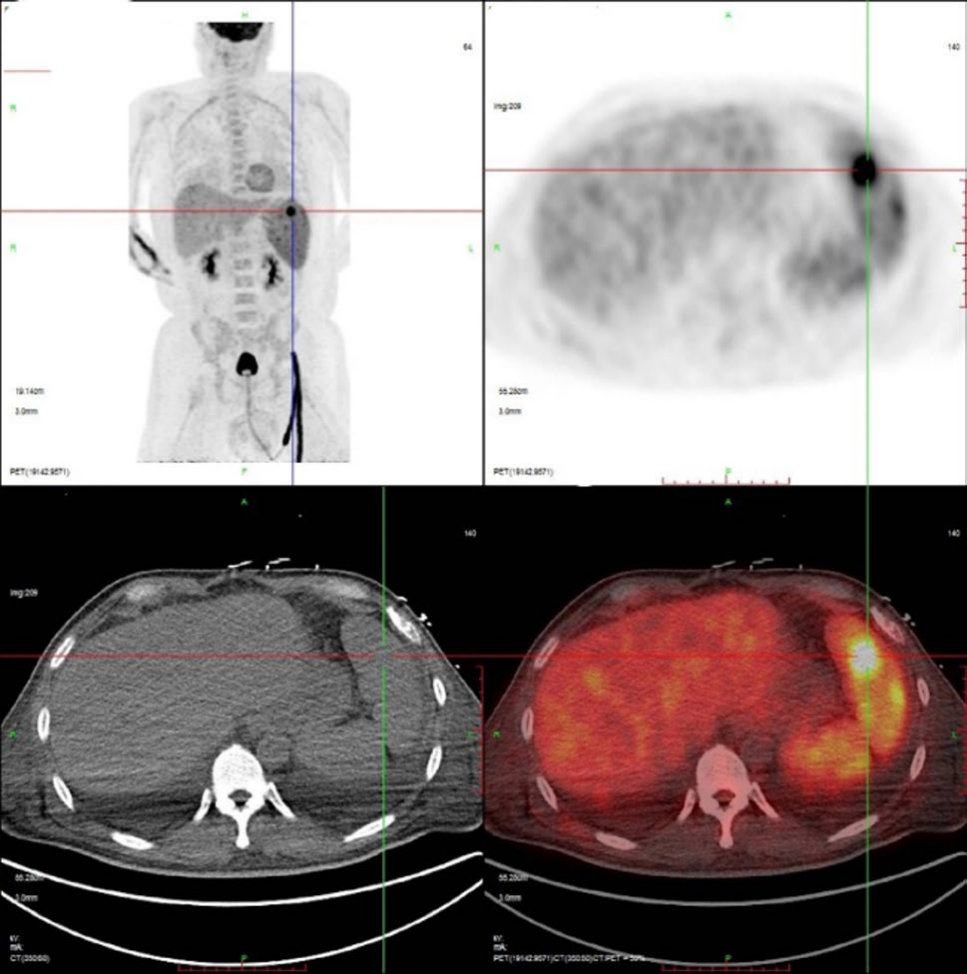
PET-CT imaging demonstrates splenomegaly with focal heterogeneous FDG uptake (SUVmax 9.0) in an area measuring approximately 3.0 × 2.0 cm. The elevated SUVmax indicates active lymphomatous infiltration and confirms extranodal involvement

**Fig. 3:**
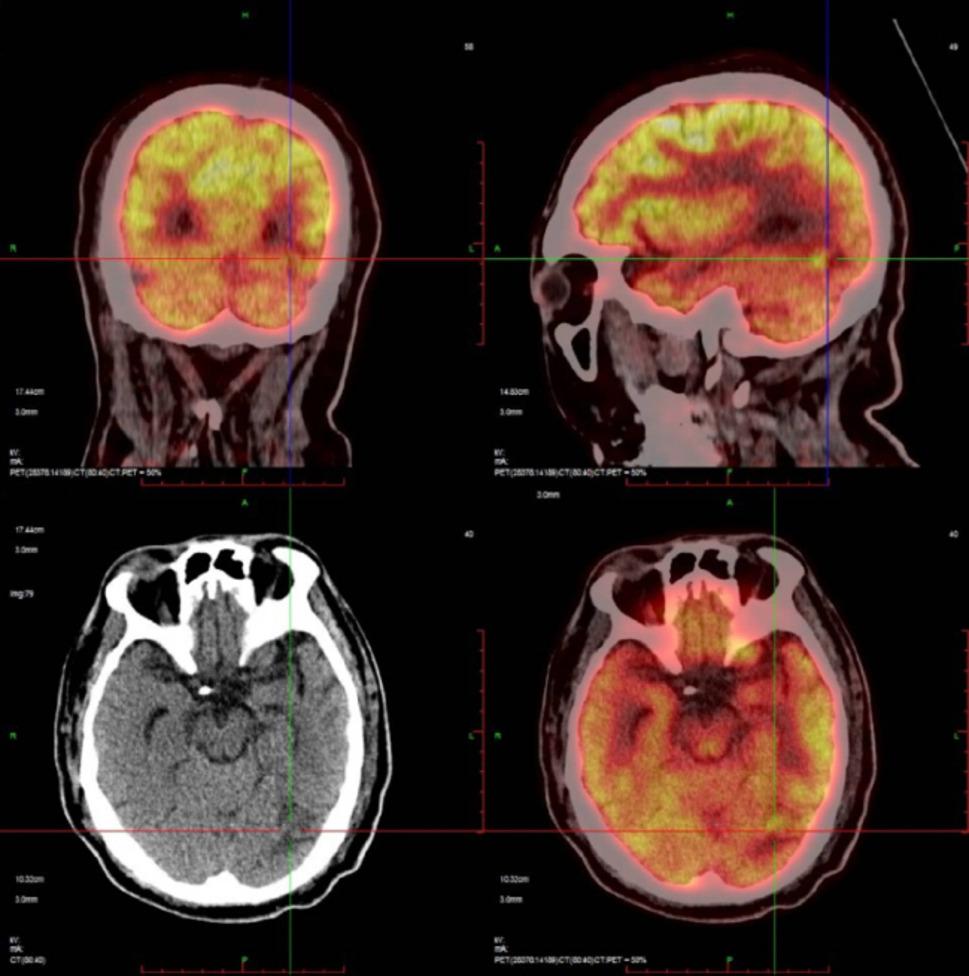
PET-CT imaging demonstrates a hypodense lesion in the left occipital lobe (arrows), representing CNS involvement. This finding correlates with the patient’s visual hallucinations and indicates advanced disease

A previously healthy 51-year-old Chinese male presented to the emergency department with an 8-day history of progressive dyspnea, high-grade fever (up to 39 °C), cough, and palpitations. Initial assessment revealed severe hypoxemia (SpO₂ 70–90%) and bilateral pulmonary infiltrates with pleural effusions on chest computed tomography (Fig. 1). Despite empirical antibiotic therapy with levofloxacin and ceftriaxone, his condition rapidly deteriorated, necessitating transfer to the emergency intensive care unit with escalating oxygen requirements (10 L/min, SpO₂ 94%). Physical examination revealed persistent tachycardia (119 bpm), tachypnea (26/min), a large ecchymosis (20 × 10 cm) on the right flank, bilateral decreased breath sounds with crackles, lower extremity edema, and palpable splenomegaly. Laboratory investigations demonstrated pancytopenia (WBC 3.26 × 10⁹/L, hemoglobin 105 g/L, platelets 58 × 10⁹/L), coagulopathy (PT 16.1 s, INR 1.5, fibrinogen 0.63 g/L), hepatic dysfunction (ALT 147 U/L, AST 154 U/L), hypoalbuminemia (24.1 g/L), and markedly elevated lactate dehydrogenase (773 U/L). Procalcitonin remained low (< 0.1 ng/mL), and pulmonary embolism was excluded by CT angiography. Thoracentesis revealed pink-tinged, turbid fluid consistent with chylothorax (triglycerides 1.46 mmol/L, positive Sudan III staining), with predominantly mononuclear cells (95%) but negative cytology for malignancy. Additional studies showed elevated ferritin (596 mg/L), bilateral cervical and supraclavicular lymphadenopathy (maximum 2.7 × 0.8 cm), and splenomegaly (4.3 cm below costal margin) on ultrasound. Bone marrow examination revealed active hyperplasia with megakaryocyte proliferation but no morphologic evidence of malignancy. However, flow cytometry analysis across three compartments revealed widespread clonal NK-cell proliferation. Peripheral blood analysis showed markedly elevated NK cells (CD16/CD56+) comprising 68.09% of total lymphocytes (normal range 6–38%) with an absolute count of 820/µL (normal 84–724/µL), accompanied by profound suppression of T-cell populations: CD3 + 7.04% (normal 50–82%), CD4 + 6.18% (normal 24–54%), CD8 + 0.90% (normal 14–41%), and B cells (CD19+) 2.19% (normal 5–21%). Bone marrow flow cytometry demonstrated 13.75% abnormal mature NK cells characterized by expression of CD16, CD56, CD94, granzyme B, HLA-DR, and CD38, with aberrant loss of CD7, and negativity for CD3, CD4, CD8, CD5, CD57, CD161, CD158a, CD158b, CD158e, and CD34. Pleural fluid flow cytometry revealed the highest tumor burden, with abnormal NK cells comprising 87.34% of nucleated cells. These cells expressed CD56, CD94, CD2, HLA-DR, and CD38, while negative for CD7, CD57, CD3, CD4, CD8, CD5, CD161, CD158a, CD158b, CD158e, NKG2A, NKG2C, and notably CD16 (in contrast to bone marrow and peripheral blood findings). Immunohistochemical analysis of pleural fluid sediment confirmed diffuse strong expression of CD2, CD56, TIA-1, and granzyme B, with negative for CD3, CD4, CD5, CD7, CD8, and CD20. In situ hybridization for Epstein-Barr virus-encoded RNA (EBER-ISH) was negative. Tissue biopsy of lymph nodes or spleen was not pursued due to severe coagulopathy (fibrinogen 0.63 g/L, INR 1.5, platelets 58 × 10⁹/L), hemodynamic instability requiring high-flow oxygen support, and rapid clinical deterioration precluding invasive procedures.These findings established the diagnosis of mature NK-cell lymphoma with peripheral blood involvement and pleural dissemination. Extensive infectious workup was negative, including Epstein-Barr virus by both serology (EBV VCA IgM/IgG) and polymerase chain reaction, cytomegalovirus, and comprehensive respiratory pathogen panel. The patient’s clinical course was complicated by progressive anxiety and emotional lability, culminating in acute hyperactive delirium on hospital day 10, characterized by visual hallucinations, paranoid delusions regarding medical staff, and agitated behavior requiring sedation and family intervention. Subsequent PET-CT imaging revealed splenic involvement (SUVmax 9.0) (Fig. 2) and a hypodense lesion in the left occipital lobe (Fig. 3), supporting the diagnosis of mature NK-cell lymphoma with central nervous system involvement. Despite supportive care, the patient rapidly progressed to hemophagocytic lymphohistiocytosis with multi-organ failure and died four weeks after presentation.

## Discussion and conclusions

Chylothorax results from thoracic duct disruption, with lymphomas comprising approximately 70% of malignancy-associated cases [11–13]. The pathogenesis involves both mechanical and biological mechanisms. Direct lymphomatous infiltration of mediastinal lymph nodes or thoracic duct invasion causes mechanical obstruction of lymphatic drainage [13]. Additionally, tumor-secreted cytokines including vascular endothelial growth factor (VEGF) increase lymphatic permeability and promote fluid accumulation [12]. In our patient, the absence of FDG uptake within the pleural effusion on PET-CT (Fig. 1) indicates lymphatic obstruction rather than malignant pleural involvement, distinguishing mechanical chylothorax from malignant effusion. The atypical presentation in our patient, featuring bloody rather than characteristically milky pleural fluid, initially obscured the diagnosis and exemplifies the diagnostic challenges encountered in emergency settings. Uncommon non-malignant etiologies must also be considered in the differential diagnosis [14].

The concurrent development of hemophagocytic lymphohistiocytosis in our patient, evidenced by fever, splenomegaly, cytopenias, coagulopathy, and elevated ferritin levels, provided a crucial diagnostic clue. Malignancy-associated HLH occurs in approximately 50% of adult HLH cases, with hematologic malignancies representing the predominant trigger [9]. The combination of chylothorax and HLH should prompt immediate consideration of underlying lymphoproliferative disorders, particularly in emergency department settings where such presentations may initially masquerade as sepsis. The neuropsychiatric manifestations in our patient warrant detailed discussion. Alternative causes of delirium were systematically excluded: metabolic encephalopathy (normal ammonia, electrolytes, renal function), septic encephalopathy (negative cultures, low procalcitonin < 0.1 ng/mL), hypoxic encephalopathy (adequate oxygenation SpO₂ >92%), and medication-induced delirium (no psychoactive agents). CNS infection could not be definitively excluded due to contraindications to lumbar puncture. The pathophysiology of delirium in our patient likely involved dual mechanisms. The hypodense lesion in the left occipital lobe on PET-CT (Fig. 3) indicates direct parenchymal infiltration, which anatomically correlates with visual hallucinations, as the occipital cortex processes visual perception [15]. Additionally, the concurrent hemophagocytic lymphohistiocytosis contributed through cytokine-mediated mechanisms. Elevated pro-inflammatory cytokines (IL-6, TNF-α, IL-1β, IFN-γ) characteristic of HLH can disrupt the blood-brain barrier and cause encephalopathy independent of direct CNS infiltration [16, 17]. This combination of direct tumor effects and systemic inflammation explains the acute hyperactive delirium phenotype. Central nervous system involvement in extranodal NK/T-cell lymphoma occurs infrequently, with reported rates varying by disease stage and risk factors [18]. The neurologic manifestations typically present as focal lesions rather than diffuse encephalopathy, making our patient’s presentation particularly unusual.

The EBV-negative status in our case warrants particular emphasis, as it represents a rare variant of extranodal NK/T-cell lymphoma. Epstein-Barr virus is detected in 80–95% of these malignancies and plays a central pathogenic role through expression of latent membrane proteins (LMP1, LMP2A) and constitutive activation of NF-κB and JAK-STAT pathways [19, 20]. EBV-negative cases constitute a rare subset, accounting for only 5–10% of NK/T-cell lymphomas, with potentially distinct molecular pathogenesis. EBV-negative cases harbor distinct genetic alterations (*STAT3/STAT5B*, *DDX3X*, *TP53* mutations) [21, 22], potentially explaining the atypical dissemination pattern observed in our case. Emerging evidence suggests EBV-negative cases may exhibit different clinical behavior and responses to targeted therapies, though limited data preclude definitive conclusions [23]. Extranodal NK/T-cell lymphoma demonstrates marked geographic variation, with significantly higher incidence rates in East Asian populations [3]. These malignancies exhibit aggressive clinical courses with poor prognosis, particularly in cases presenting with extranasal involvement. The median overall survival for patients with non-nasal NK/T-cell lymphoma ranges from 6 to 12 months, with many patients surviving only for weeks to months following diagnosis [23].

Several clinical considerations merit emphasis: chylothorax may present atypically with bloody fluid, necessitating biochemical confirmation; combined pleural and neuropsychiatric symptoms warrant malignancy investigation; and flow cytometry provides value when cytology is non-diagnostic.

Several limitations warrant acknowledgment. Tissue histopathology was not obtained due to severe coagulopathy (fibrinogen 0.63 g/L, platelets 58 × 10⁹/L) and hemodynamic instability, precluding safe biopsy. While WHO classification emphasizes histologic confirmation [1], the diagnosis was supported by characteristic immunophenotype across multiple sites, PET-CT findings, clinical course, and exclusion of alternative diagnoses. Additionally, lack of cytogenetic and molecular studies (such as *STAT3/STAT5B* mutations, *DDX3X *alterations) limits understanding of pathogenic mechanisms in this EBV-negative variant.

This novel presentation of chylothorax and delirium in NK-cell lymphoma expands the recognized emergency spectrum. When encountering unexplained pleural effusion with neuropsychiatric symptoms and cytopenias, emergency physicians should maintain high suspicion for hematologic malignancy and pursue prompt hematology consultation. Despite poor prognosis, timely diagnosis enables appropriate care decisions.

## Data Availability

The data generated in the present study may be requested from the corresponding author.
